# Synthetic Lethality-based Identification of Targets for Anticancer Drugs in the Human Signaling Network

**DOI:** 10.1038/s41598-018-26783-w

**Published:** 2018-05-31

**Authors:** Lei Liu, Xiujie Chen, Chunyu Hu, Denan Zhang, Zhuo Shao, Qing Jin, Jingbo Yang, Hongbo Xie, Bo Liu, Ming Hu, Kehui Ke

**Affiliations:** College of Bioinformatics Science and Technology, Harbin Medical University, 194 Xuefu Road, Harbin, 150081 China

## Abstract

Chemotherapy agents can cause serious adverse effects by attacking both cancer tissues and normal tissues. Therefore, we proposed a synthetic lethality (SL) concept-based computational method to identify specific anticancer drug targets. First, a 3-step screening strategy (network-based, frequency-based and function-based screening) was proposed to identify the SL gene pairs by mining 697 cancer genes and the human signaling network, which had 6306 proteins and 62937 protein-protein interactions. The network-based screening was composed of a stability score constructed using a network information centrality measure (the average shortest path length) and the distance-based screening between the cancer gene and the non-cancer gene. Then, the non-cancer genes were extracted and annotated using drug-target interaction and drug description information to obtain potential anticancer drug targets. Finally, the human SL data in SynLethDB, the existing drug sensitivity data and text-mining were utilized for target validation. We successfully identified 2555 SL gene pairs and 57 potential anticancer drug targets. Among them, CDK1, CDK2, PLK1 and WEE1 were verified by all three aspects and could be preferentially used in specific targeted therapy in the future.

## Introduction

Synthetic lethality (SL) was first defined by Calvin Bridges in 1922^1^, who noticed that some combinations of gene mutations in the model organism *Drosophila melanogaster* conferred lethality. This term now refers to the genetic interaction between two or more genes where only their co-alteration (e.g., by mutations, amplifications or deletions) can result in severe loss of viability or death of the cell, although the cell remains viable when the individual genes are altered^2^. The term “SL” was coined in 1946 by Theodosius Dobzhansky, who was a geneticist and evolutionary biologist and described a lethally genetic interaction as when two independently viable homologous chromosomes were allowed to recombine in *Drosophila pseudoobscura*^3^. In 1997, Hartwell *et al*. first proposed to apply the concept of SL and used chemical and genetic screening methods to develop selective anticancer drugs and anticancer drug targets^4^. Since then, SL has become a valuable concept that has led to an innovative approach for identifying specific anticancer drug targets^5,6^.

Serious adverse drug reactions are some of the main problems with cancer treatment. Conventional cancer chemotherapy that does not exploit the genetic differences between cancer tissues and normal tissues tends to produce toxic effects on normal cells. To solve the problem, targeted therapy has emerged as a hot spot in anticancer drug research and development. In addition, the discovery of “SL” creates new hope in discovering an anticancer drug target for targeted therapeutics^7^. Cancer is caused by the inactivation or mutation of particular genes in normal cells. If specific mutant genes are involved in cancer, it is possible to specifically kill cancer cells without harming healthy cells by inhibiting the SL partner gene with anticancer drugs. Even if the distribution of the SL partner gene is not specific, it will not cause a serious impact on normal cells according to the concept of SL. A major breakthrough in the targeted therapy of BRCA1-mutant cancers was the finding that cells with BRCA1/2 mutations were exquisitely sensitive to poly (ADP-ribose) polymerase (PARP) inhibitors^8,9^, which was a great utility of SL. In addition, targeted therapy achieved a milestone success via the targeting of the PARP-1 enzyme by Olaparib in ovarian cancer patients carrying a tumor BRCA1/2 mutation^10,11^.

To identify SL interactions that could be efficacious in treating cancer, many approaches have been proposed. Current screening methods for potential SL gene pairs can be summarized in three categories. The first is based on model organisms (such as yeast or fruit flies). Their genomes are small and can be easily mutated and matched; therefore, gene silencing techniques are easier to conduct in model organisms. However, as with the homologous inference methods of all model organisms, most genes in SL gene pairs in model organisms do not have homologous genes in human genome. Even though homologous genes can be found in the human genome, their functions have undergone great changes and cannot be directly converted into SL gene pairs^12^. The second screening method was gene silencing in mammals, and two types of gene silencing methods have been developed. One is based on the priori knowledge speculation^13^. The potential SL gene pairs contained two kinds of genes, namely, mutant cancer genes and SL partner genes. Therefore, the SL partner genes should be directly knocked down and tested one by one. The other is based on high-throughput experimental techniques for unbiased screening of the whole genome^14^. Ultimately, siRNA and CRISPR screenings proved to be the most reliable methods for detecting SL gene pairs^15^. However, compared to model genetic systems, human cell systems face greater challenges for genome-wide siRNA or CRISPR screening. Moreover, these approaches are considerably more expensive, labor-intensive, time consuming and many of the essential genes so identified turn out to be either restricted to only these cell-line models or are in frequently overexpressed in cancers^16^. For these reasons, the third screening method based on computational methods has attracted more and more attention.

Computational approaches, which can help to identify and prioritize potential SL gene pairs for further experimental validation, represent an attractive alternative compared to genome-wide siRNA or CRISPR-based human cell line screening approaches. These methods include human orthologous gene pairs inference from yeast SL genes^7,17^; the use of robustness features in the cancer PPI network to evaluate the importance of gene pairs^18^; a mutual exclusivity calculation using statistical models from gene mutation/transcriptional expression data^19,20^; data-driven detection of SL (DAISY) that combined somatic copy number alteration, siRNA screening and cell survival and gene co-expression information and achieved a promising performance in data-driven SL gene pair identification^21^; and a learning-based pipeline for training and prediction, which combined the three features of mutation coverage, driver mutation probability and network information centrality into a manifolds ranking model to generate a ranking list of potential SL pairs^16^.

Furthermore, the methods mentioned above are not based on the human biological system or cannot be a good simulation of the human complex and staggered environments. The cells employ signaling pathways and networks to drive biological processes in which genomic alterations might result in malignant signaling, which then leads to cancer phenotypes^22^. In this article, the human system was abstracted into a human signaling network. The specific mutant gene was defined as a cancer gene and its SL partner gene was defined as the non-cancer gene. Then, we proposed a computational method using a 3-step screening strategy to identify SL gene pairs from the perspective of a network system. Next, according to the SL gene pairs we identified, we extracted non-cancer genes to obtain anticancer drug targets. Finally, we used 3 different aspects of data to validate parts of our results. Overall, the SL strategy contributes to the identification of anticancer drug targets and drug redirection.

## Results

### Human cancer signaling network

**Figure 1:**
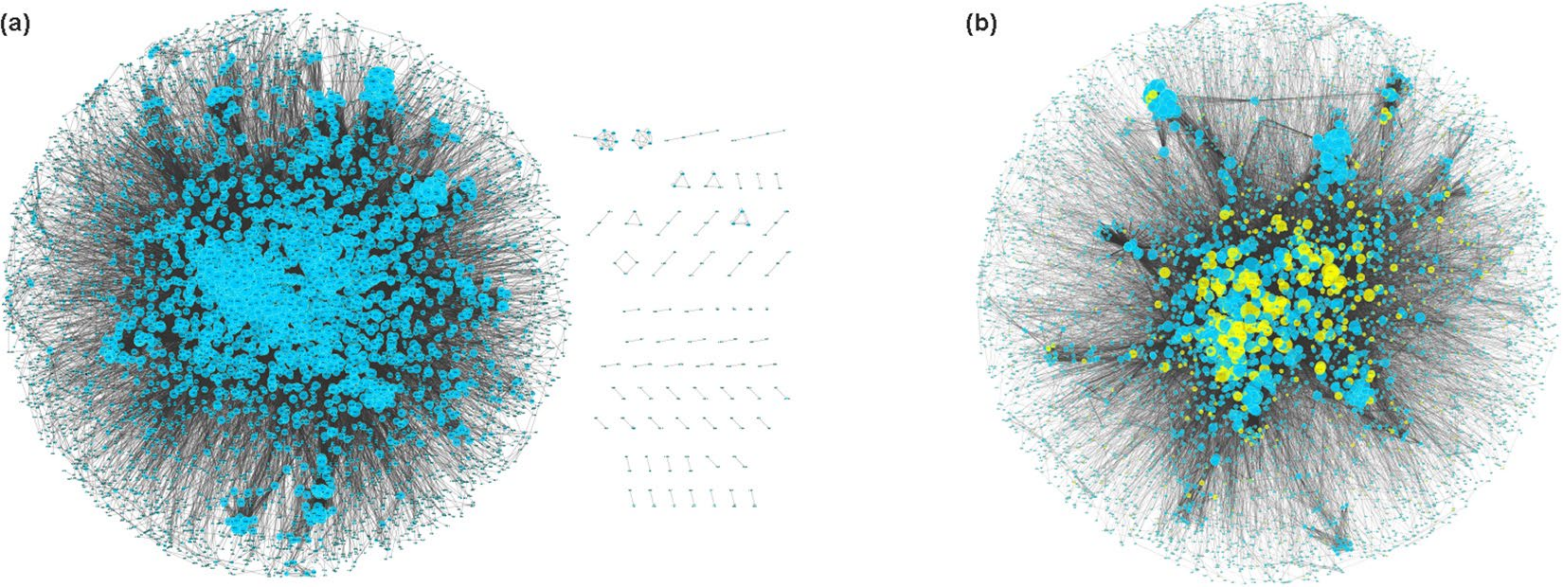
The illustration of the network. (**a**) The human signaling network. (**b**) The human cancer signaling network (HCSN). Blue nodes denote non-cancer genes; yellow nodes denote cancer genes; and edges represent protein-protein interactions. A larger node indicates a greater degree.

This subject focused on high-frequency non-cancer genes that have a greater impact on biological systems. Thus, the frequencies of all non-cancer genes were counted according to the genes passing through the shortest path between all cancer gene and non-cancer gene pairs in the human signaling network (Fig. 1(a)). All of the nodes in the human signaling network were sorted by frequency in descending order. Then, the top 30% (740) of non-cancer genes were obtained to construct a network named the human cancer signaling network (HCSN) for further research. As shown in Fig. 1(b), HCSN includes 6153 proteins and 56976 protein-protein interactions, and 697 cancer genes were successfully mapped. Thus, non-cancer genes were paired with cancer genes to form 515780 (740 × 697) gene pairs, which were used as input data for the following 3-step screening strategy for identifying SL gene pairs.

### SL gene pairs

We designed a 3-step screening strategy to predict the SL gene pairs in the HCSN, and the results are described herein.

First, we chose the network-based screening method to obtain the SL gene pairs. According to the stability score and 1000 randomized networks (P < 0.05), we obtained the significant SL gene pairs. Then, we screened the gene pairs based on the distance between non-cancer genes and cancer genes. The average distance between non-cancer gene and cancer gene was 2.90; therefore, we kept the gene pairs with distances no more than 2. After the first screening step, 9241 gene pairs were obtained.

**Figure 2:**
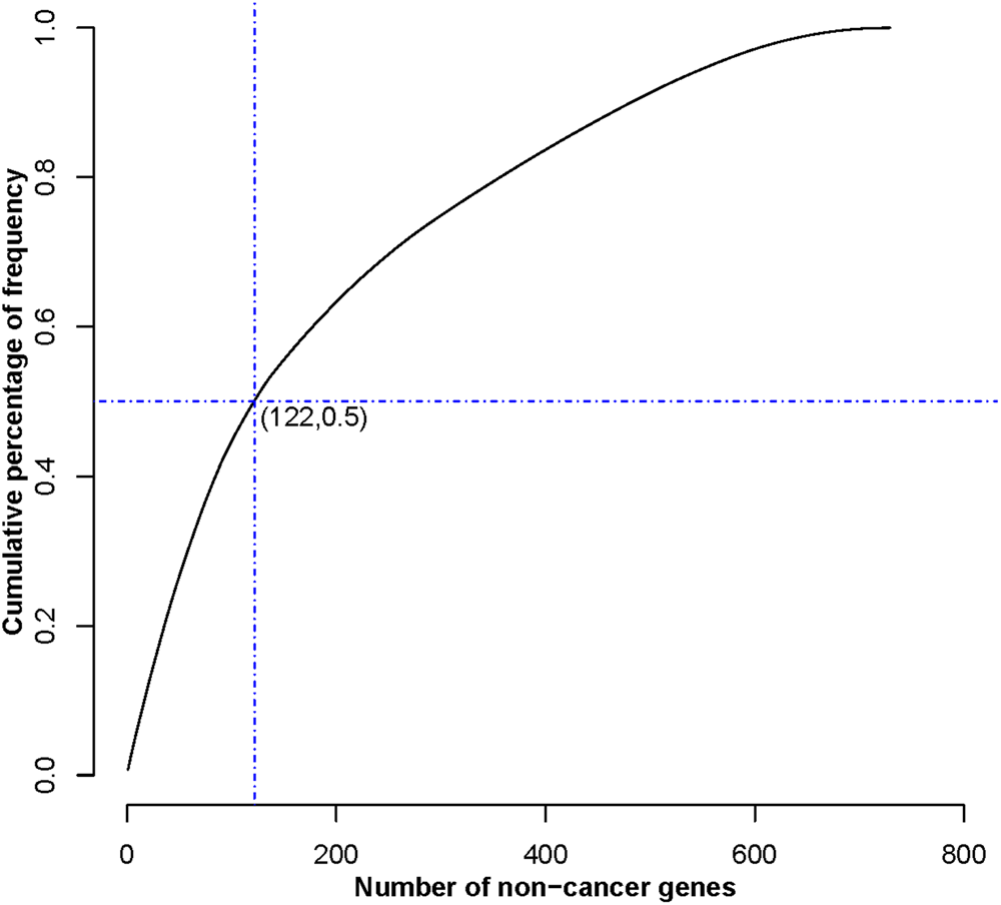
The cumulative percentage of frequency. The X-axis was the number of non-cancer genes. The Y-axis was the cumulative percentage of frequency. (122, 0.5) represented the cumulative frequency of the first highly frequent 122 genes account for 50% of the cumulative frequency of the total genes.

Second, we chose the frequency-based screening method. We plotted the cumulative frequency percentage plot to obtain a reasonable frequency threshold (Fig. 2). As seen from the figure, the growth trend of the top 50% curve was faster. Therefore, 122 high-frequency non-cancer genes were focused on in our study. As a result, 4788 gene pairs were obtained.

**Figure 3:**
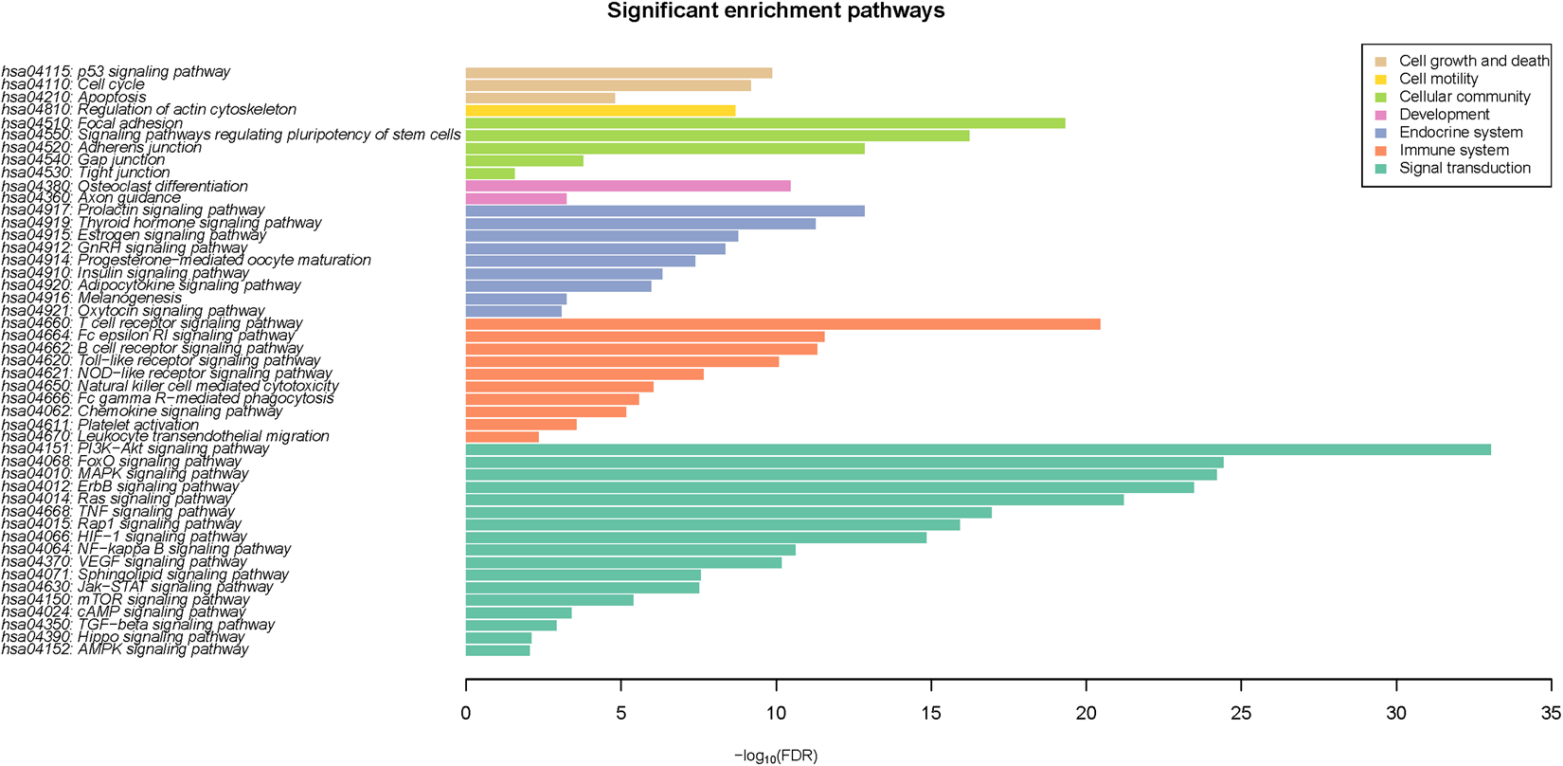
The significant enrichment pathways. Different colors denoted different pathway categories.

Third, the function-based screening method was performed. The 4788 gene pairs from the second screening contained 749 genes and these genes were significantly enriched in 47 pathways (Fig. 3). These pathways could be divided into seven biological process categories, namely, cell growth and death, cell motility, signal transduction, endocrine system, immune system, cell community and growth. Many biological pathways in our results were found to be closely related to SL. For example, the HIF-1 signaling pathway, which activated the transcription of genes involved in angiogenesis, cell survival, glucose metabolism and invasion, was used as a screening pathway for the discovery of SL gene pairs^23^. The PI3K-AKT signaling pathway^24^, the RAS signaling pathway^25^, the P53 signaling pathway^26^, and the mTOR signaling pathway^27^ were also widely considered promising pathways for SL recognition and have attracted the interest of many researchers.

**Figure 4:**
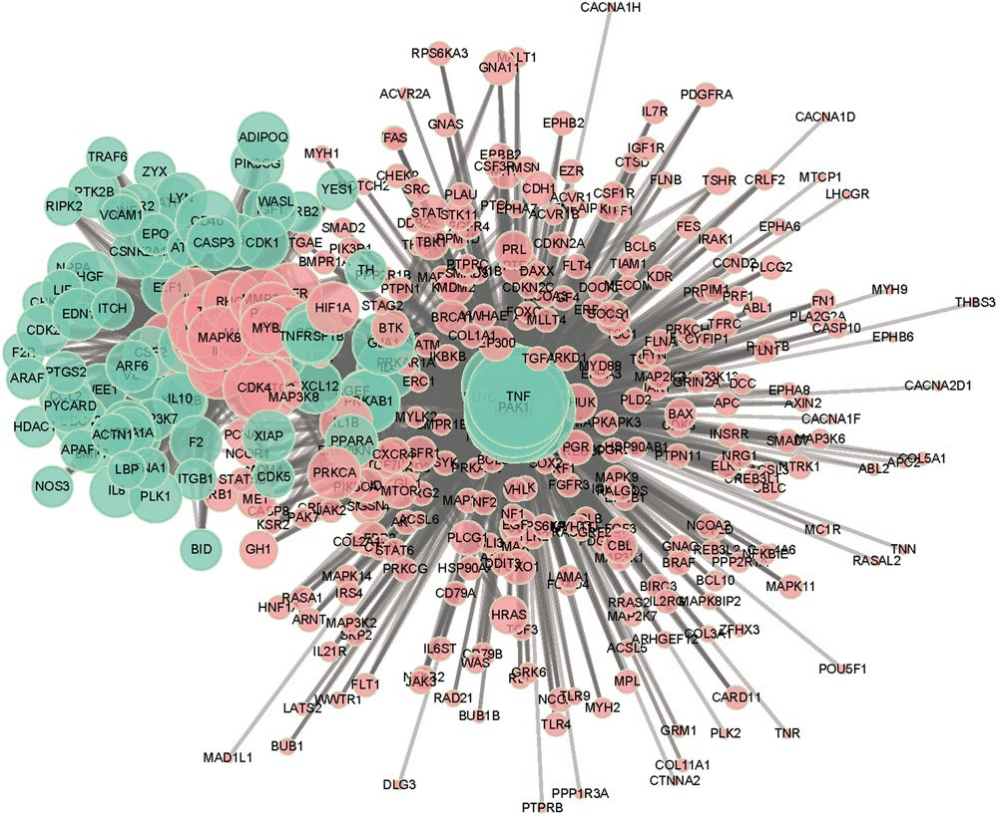
SL gene pairs. Light blue nodes denoted non-cancer genes; red nodes denoted cancer genes. Larger node indicates greater degree.

In addition, after function-based screening, we obtained 395 significantly enriched genes conformed 2555 SL gene pairs, which included 81 non-cancer genes and 314 cancer genes (Fig. 4). The average degree of the light blue nodes and red nodes were 8.14 and 31.54, respectively. According to the concept of SL, we think that these 81 non-cancer genes should be potential and specific anticancer drug targets. Designing drugs against these non-cancer genes in cancer with specific cancer gene mutations could improve the therapeutic efficiency and reduce side effects. However, at the same time, many aspects need to be considered before a protein that could be used as a drug target such as molecular weight, polarity, and tissue distribution in the body. Therefore, we focused on the existing drug target information and our non-cancer genes in the SL gene pairs to explore adaptive anticancer drug targets.

### Potential anticancer drug targets

We used the existing drug-target interaction data and 81 non-cancer genes in SL gene pairs to extract specific anticancer targets and drugs, which might be used in cancer treatment. After we annotated the 81 non-cancer genes with the drug-target information, 57 known drug targets (Table 1) were identified, of which 27 had been used as anticancer drug targets in clinical treatment. Using these 27 targets, we expected specific and low-risk cancer therapies to be achieved. In our opinion, the rest of the 30 targets, which are closely related to the occurrence and progression of cancer, such as immune-related and anti-inflammatory targets, have the potential to become anticancer drug targets and will be used in anticancer drug re-positioning.

**Table 1:** The potential anticancer targets and corresponding non-cancer target.

Non-cancer Gene	Target	Target Type	Non-cancer Gene	Target	Target Type
CCL2	P13500	Anticancer drug target	IL18	Q14116	Anti-Inflammatory target
CDK1	P06493	Anticancer drug target	VCAM1	P19320	Anti-Inflammatory target
CDK2	P24941	Anticancer drug target	CD4	P01730	Immune-related target
CDK5	Q00535	Anticancer drug target	CSF2	P04141	Immune-related target
CSF1	P09603	Anticancer drug target	GRB2	P62993	Immune-related target
CSNK2A1	P68400	Anticancer drug target	IL10	P22301	Immune-related target
E2F1	Q01094	Anticancer drug target	ITGB1	P05556	Immune-related target
F2	P00734	Anticancer drug target	TH	P07101	Immune-related target
FGF2	P09038	Anticancer drug target	APAF1	O14727	other
HDAC1	Q13547	Anticancer drug target	ARAF	P10398	other
HGF	P14210	Anticancer drug target	ARF6	P62330	other
IL6	P05231	Anticancer drug target	ATF2	P15336	other
LYN	P07948	Anticancer drug target	ATF4	P18848	other
MAPK3	P27361	Anticancer drug target	BDNF	P23560	other
MMP9	P14780	Anticancer drug target	CASP3	P42574	other
NFKB1	P19838	Anticancer drug target	CD40	P25942	other
NOS3	P29474	Anticancer drug target	CDC42	P60953	other
PRKCZ	Q05513	Anticancer drug target	CXCL12	P48061	other
PTGS2	P35354	Anticancer drug target	EDN1	P05305	other
PTK2B	Q14289	Anticancer drug target	F2R	P25116	other
TNF	P01375	Anticancer drug target	GJA1	P17302	other
TNFRSF1B	P20333	Anticancer drug target	IGF1	P05019	other
VEGFA	P15692	Anticancer drug target	INS	P01308	other
XIAP	P98170	Anticancer drug target	KAT2B	Q92831	other
YES1	P07947	Anticancer drug target	NPR2	P20594	other
PLK1	P53350	Anticancer drug target	NPY	P01303	other
WEE1	P30291	Anticancer drug target	PIK3CG	P48736	other
		Anti-Inflammatory target;	SGK1	O00141	other
PPARA	Q07869	Analgesics drug target;	PRKAB1	Q9Y478	Analgesics drug target
		Immune-related target			Anti-Inflammatory target;

In addition, the average degree of the 57 drug targets was 33.81, which indicates those nodes had interactions with more red nodes in the network (Fig. 4). Meanwhile, some light blue nodes showed a large degree, but they weren’t known drug targets such as PAK1 and IL4. The frequencies of PAK1 and IL4 were 269 and 60, respectively. PAK1 encodes a family member of the serine/threonine p21-activating kinases, also known as the PAK proteins. This specific family member regulates cell motility and morphology. In addition, PAK1 could be mapped into many promising SL recognized pathways such as the MAPK signaling pathway, focal adhesion, and the ErbB signaling pathway. The protein encoded by the IL4 gene is a pleiotropic cytokine produced by activated T cells. This cytokine is a ligand for the interleukin 4 receptor. In addition, it could be mapped into the T cell receptor signaling pathway and the Fc epsilon RI signaling pathway. Therefore, those light blue nodes that had large degree also tend to have great effects in specific anticancer therapy in combination with the SL gene pairs we identified.

### Validation of the anticancer drug target

**Figure 5:**
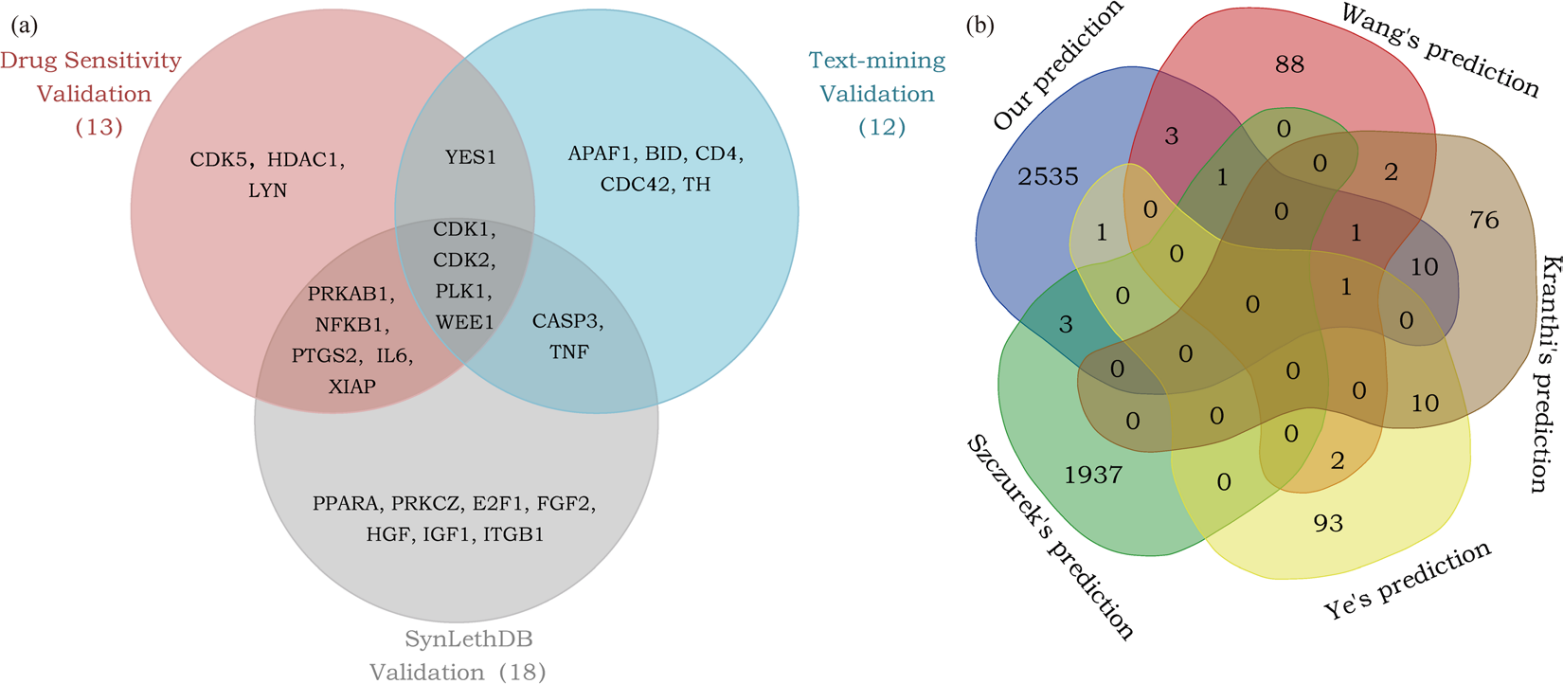
Illustration of our validations. (**a**) Anticancer drug targets validated by three aspects of the data. In the SynLethDB validation, drug sensitivity validation and text-mining validation, we validated 18, 13 and 12 anticancer drug targets, respectively. In addition, 4 targets could be validated using all three aspects. (**b**) The Venn diagram was drawn based on the overlap of the predicted SL gene pairs in four previous reports and our results. The methods with extremely low concordance of the results are not shown in the figure, which was drawn with the online tool http://bioinformatics.psb.ugent.be/webtools/Venn/.

To verify the results, three aspects of the data were used. The first was SynLethDB^28^, which contained SL pairs information collected from biochemical assays, computational predictions, text mining results and other related databases. We used the overlap gene data between SynLethDB and our predicted anticancer drug targets information to validate the results. Because of the limitation of the SL gene pair data, 20 of the 57 known drug targets that we found were not included in SynLethDB. As a result, 18 of the 37 anticancer drug targets were validated as SL partner genes in this database. These targets with corresponding cancer genes constitute 35 SL gene pairs in our predicted results (Supplementary Table S1). The second was the known drug sensitivity data. Among the data of drug targets that was used, 13 were overlapped with our result. In different cancer cell lines, a smaller IC50 value indicates higher drug sensitivity and the corresponding drug target tends to have better effects in cancer therapy. More information is shown in Supplementary Table S2 (only IC50 values less than 0 are shown). Finally, we conducted text-mining to determine the relationship between the anticancer drug targets that we found and the genes related to cancer (or SL). The results showed that 52 of the 81 non-cancer genes had been shown to be significantly associated with cancer (p < 0.05) and 16 of the 81 non-cancer genes had been shown to be significantly correlated with SL. Furthermore, 12 anticancer drug targets were closely associated with both SL and cancer (see Supplementary Table S3). In total, 27 of the 57 anticancer drug targets were verified through three different aspects and 4 targets have been verified in all three aspects of the data, as shown in Fig. 5(a).

In particular, the four overlap non-cancer genes (CDK1, CDK2, PLK1 and WEE1), which were validated by the three data resources, were all known anticancer drug targets and clinical trial targets. Furthermore, CDK1, CDK2, PLK1 and WEE1 were also predicted to be promising anticancer targets in BRCA2-ovarian cancers by Bueno’s research^29^. Therefore, we focused on the analysis of these four overlap genes. Above all, the CDK1 and CDK2, which can be promising specific anticancer target, are both family members of the serine/threonine protein kinases that participate in cell cycle regulation. Firstly, CDK1 can be the SL partner gene of the cancer genes KRAS and MYC. As reported, KRAS mutations have been found in approximately 20% of human cancers, but there is currently no therapy targeting them^30^. Thus, targeting the SL partner gene CDK1 in ovarian cancer patients carrying a KRAS mutation could be a good choice in anticancer drug research and development. Although the cancer gene MYC is a very attractive therapeutic target in the treatment of breast cancer, the direct inhibition of the MYC gene is still a great challenge and has not yet provided a clinically effective drug to target it^31^. In the MYC-dependent breast cancer, another alternative is to target MYC’s SL partner gene CDK1, as reported in some small interfering RNA (siRNA) experiment^31^. Secondly, CDK2 was predicted to be SL partner gene with p53 and MYCN by RNA interference techniques^32,33^. In p53 defective cells, CDK2 can separate mitogenic from anti-apoptotic signaling for SL^33^. The SL relationship between CDK2 and MYCN indicates CDK2 inhibitors as potential MYCN-selective cancer therapeutics^32^. Furthermore, CDK1 and CDK2 are both drug targets of the investigational drug Alvocidib which is a synthetic flavonoid based on an extract from an Indian plant for the potential treatment of cancer. It works by inhibiting CDK, arresting cell division and causing apoptosis in non-small lung cancer cells^34^. According to the concept of SL, using Alvocidib to target CDK1 may selectively kill specific gene mutant tumor cells. Then, PLK1, which was a drug target studied in acute myeloid leukemia, non-small cell lung cancer, and pancreatic cancer^34^, could be a SL partner gene of many cancer genes in our results. In the drug sensitivity validation, some cells are sensitive to the drug target PLK1, which indicates that PLK1 can participate in various cancers by forming SL gene pairs with many cancer genes. Furthermore, some researches has identified PLK1 as a gene whose depletion was particularly detrimental to the viability of PIM1-overexpressing prostate cancer, which was particularly sensitive to PLK1 inhibition and suggests that PIM1 might be used as a marker for identifying patients who will benefit from PLK1 inhibitor treatment^35^. Finally, WEE1 kinase could regulate CDK1 and CDK2 activity to facilitate DNA replication during S-phase and prevent unscheduled entry into mitosis, and cancers with defects in the FA and HR pathways may be targeted by WEE1 inhibition, which provides a basis for a novel SL strategy for cancers harboring FA/HR defects^36^. In addition to the four intersection genes, many of the other non-cancer genes that we identified have already been predicted as the anticancer drug targets. For example, in the drug sensitivity experiment, IL6, which could be the SL partner gene of CDKN2A, RB1, STK11 and TP53, was a specific anticancer drug target in the prostate cancer DU-145 cell line when targeted by VX-702^37^.

## Discussion

With the development of molecular biology, biological research has entered the post-genome era and has made it possible to understand the function of the organism from an overall level. Synthetic biological systems (human protein interaction networks) are complex, and each protein element is a node in the complex network that accomplishes each biological process by synergizing the interactions of the nodes. Thus, the biological network can be abstractly seen as a human biological system and provides pre-screening for *in vitro* and *in vivo* follow-up anticancer drug targets screening. It can also save financial and material resources and time.

The existing approach, which also used networks to identify SL gene pairs, was proven to be effective^18^. However, they only took the efficiency changes of knocking out two nodes in the network into account. Since this change may sometimes be caused by knocking out a single gene node rather than the pair, we improved the method by considering the knockout of both a single node and two nodes, which was more reliable in our opinion. Furthermore, we took a multi-step screening strategy from many perspectives to obtain the SL gene pairs, which might get better results.

Although this study has many advantages, there are some shortcomings. The most significant one is that the data resources we used. On one hand, it is the original data we used for this study. Although we integrated the cancer gene data and drug-target interactions data from different databases, more data should be included in the future to obtain more useful results. This way, we will improve the accuracy of our results and reduce data limitations. On the other hand, it is the limitation of the validation data. The genes and drugs in the drug sensitivity experiment are relatively small, so we could only validate the overlapping genes between the existing data and our studies. The SynLethDB database, which we used to validate, included 16976 SL gene pairs composed by 5157 genes. Only 7088 SL gene pairs (7088/16976 = 41.75%) that composed by 2174 genes (2174/5157 = 42.16%) were found in our network data. At the same time, we made a comparison between all 5157 genes in SynLethDB and our 697 input cancer genes, the overlap genes were only 369 (52.94%), which constituted 8582 SL gene pairs (8582/16976 = 55.55%) in SynLethDB. As can be seen from above, the data contained in the SynLethDB were very different from our input data. As a result, we can only validate the overlap part between SynLethDB and ours. We also tried to make a comparison with other state-of-the-art computational SL finding methods. However, various computational methods provided potential SL gene pairs from different data resources and perspectives, such as the correlation of gene expression with mutations, gene co-expression in related biological processes, robustness in the cancer network or human conserved SL gene interactions, which may be the reason for the low coincidence rate of the SL gene pairs obtained from different computational methods. At the same time, we compared the 2555 predicted SL gene pairs (81 non-cancer genes and 314 cancer genes) with the results of the other seven previous computational methods^7,16,18,20,21,38,39^. As shown in Fig. 5(b), the overlap SL gene pairs of these methods was very rare (the details are shown in the Supplementary Table S4).This was not the case with our results, but also with others. The results from the different methods were complementary to each other in predicting the SL gene pairs^16^.

The 57 known drug targets that we found might be targets for anticancer drugs and could be used in drug re-positioning. Focusing on these targets can accelerate the development of anticancer drugs. The other non-cancer genes, which have not been drug targets previously, may also have potential in cancer therapy. Moreover, in different cancer cells, mutations in the same cancer gene can also lead to various functions; therefore, our follow-up study will focus on the different mutant types of the same genes, which are dedicated to finding more specific anticancer drug targets and corresponding sensitive drugs through the combination of the SL strategy.

## Materials and Methods

### Data sources

In this paper, the human signaling network, including 6306 proteins and 62937 protein-protein interactions, was collected and curated manually by Zaman^22^ from previous studies^40–42^. The cancer genes were downloaded from the F-Census^43^ and Cancer Gene Census^44^. We obtained 697 cancer genes after removing the redundant ones. Drug-target interaction data was collected from the DrugBank^45^, Therapeutic Targets Database (TTD)^34^ and PROMISCUOUS databases^46^. In addition, we obtained 16976 human SL genes pairs from the SynLethDB database^28^. The drug sensitivity data and the gene mutation backgrounds of 639 cancer cell lines were gathered from the research^37^, which contained 88 cancer genes and 130 drugs under clinical and preclinical investigation in the experiment.

### SL screening

**Figure 6:**
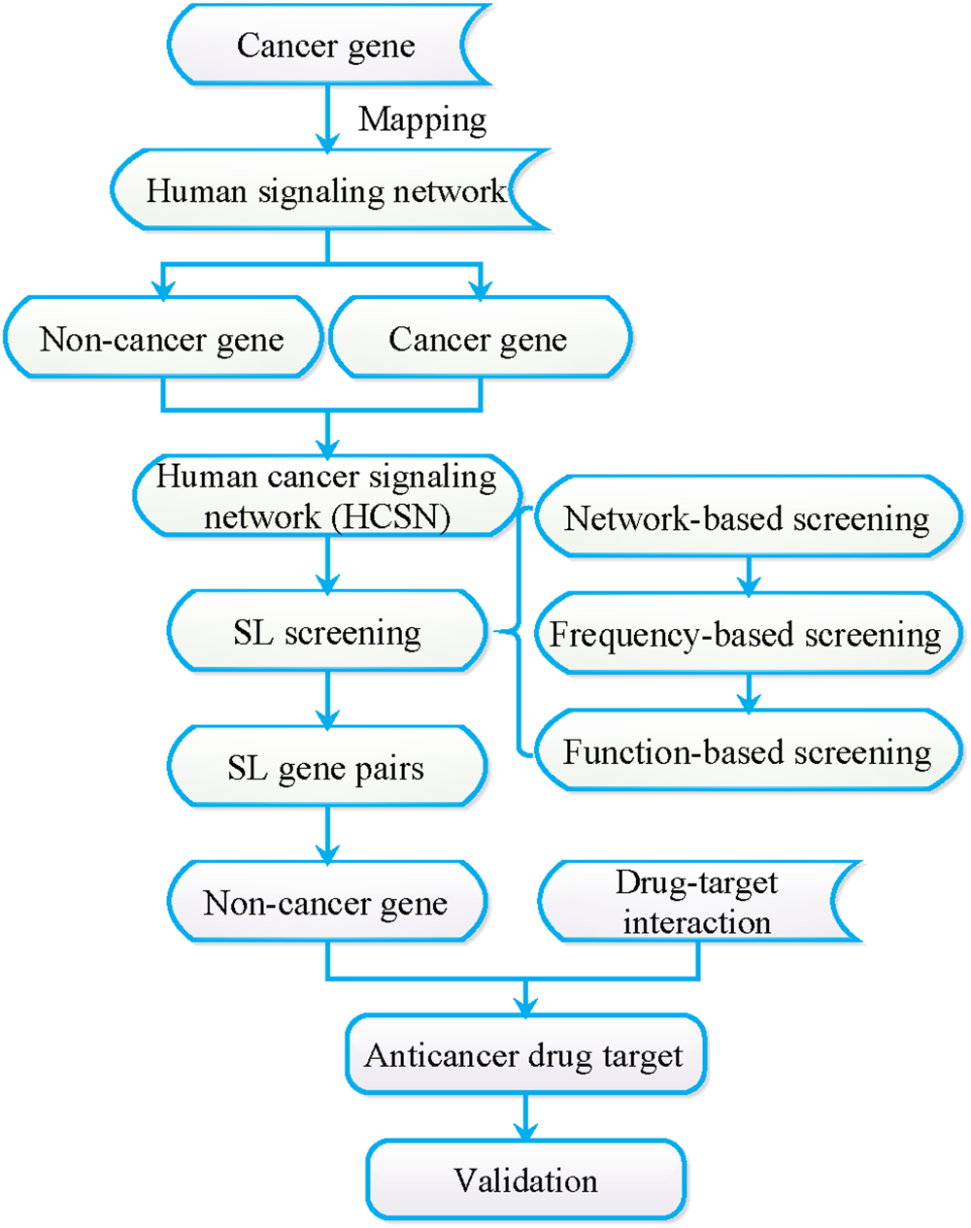
The workflow of anticancer drug targets identification. The human cancer signaling network (HCSN) was constructed to obtain SL gene pairs using a 3-step screening strategy. The data of non-cancer genes and drug-target interactions data were obtained to identify the anticancer drug targets. Some validations were made to validate our results.

The overall workflow of our method is shown in Fig. 6. Above all, we constructed the human cancer signaling network (HCSN). Next, a 3-step screening strategy was used to obtain the SL gene pairs. Then we extracted the non-cancer genes from the SL gene pairs and analyzed them with the drug-target interactions to find the targets that were suited for anticancer drugs. Finally, we conducted the validation with prior data.

#### Construction of HCSN

To get the HCSN, we removed the orphan nodes, peripheral interactions, self-loop and redundant interactions of the human signaling network and mapped the cancer genes into it. The human signaling network and HCSN could be explored using the freely available Cytoscape software (version 3.3.0)^47^. Nodes represent proteins and edges represent protein-protein interactions.

#### Obtainment of SL gene pairs

In this study, we designed a computational approach to predict SL gene pairs in the HCSN, which were mainly composed of a 3-step defined screening strategy, network-based screening, frequency-based screening and function-based screening.

### Network-based screening of gene pairs.

1. **Calculation of the stability score**
  Herein, the stability score was defined as the stability changes of HCSN when knocking out a pair of nodes and just one node. Therefore, according to the concept of SL, gene pairs, which have higher stability scores, should more likely be the SL gene pairs. A stability change may be caused by just one node rather than the combination effects of gene pairs. Therefore, we proposed a network information centrality-based approach by knocking out both a pair of nodes and the single one, respectively. Then, the network information centrality-based stability score *S* was scored in formula (1):
  1 $${S}=\frac{2{{\rm{D}}}_{m,n}-{D}_{m}-{D}_{n}}{{D}_{0}}$$
  where ***D***_**0**_ was the average shortest path length of HCSN; ***D***_***m***_ and ***D***_***n***_ represent the average shortest path length of HCSN after removing the cancer gene node *m* and the non-cancer gene node *n*, respectively; and ***D***_***m,n***_ was the average shortest path length of HCSN after removing both the cancer gene nodes ***m*** and non-cancer gene nodes ***n***. ***D*** was the average shortest path length of the network (calculated by the *closeness* in R package *igraph*^48^) and it was defined as follows in formula (2):
  2 $$D=\frac{1}{\frac{1}{2}N(N-1)}{\sum }_{i > j}{d}_{ij}$$
  here, ***d***_***ij***_ refers to the shortest path between the nodes *i* and *j*; *N* represents the total number of nodes in the network.
2. **Network randomization**
  To evaluate the significance, we calculated the probability values *p* for each of the gene pairs using 1000 degree-preserving randomized networks (constructed by R package *tnet*^49^). The formula to calculate the *P* values was as follows:
  3 $${p}=\frac{{{N}}_{{S}_{obs} < {S}_{random}}}{1000}$$
  where *S*_*obs*_ refers to the *S* score obtained from HCSN and *S*_*random*_ refers to the *S* score obtained from randomized network. ${N}_{{S}_{obs} < {S}_{random}}$ represents the numbers when the *S* score in the randomized network was larger than that in HCSN.
3. **Distance-based screening of the gene pairs**

Distance-based SL screening played a vital role in network analysis in our study. We thought that the human signaling network was very important in tumorigenesis and cancer progression. In the network, proteins next to each other may have some similar functions and will participate in certain similar biological progresses. In other words, two proteins might more likely be SL partners if they were closer in distance in the network. Therefore, we calculated the distance between every non-cancer gene and cancer gene, and then computed the average distance of those nodes. Then, we discarded the pairs for which the distance was larger than the average.

### Frequency-based screening of gene pairs

The development of cancer is often quite complex and usually involves multiple genes and pathways. We defined the nodes in HSCN with high degree as high frequency genes. We assumed that the higher frequency non-cancer genes in HCSN are more important in the biological progress. Therefore, we used the frequency of non-cancer genes as a filter for further screening. According to the cumulative frequency percentage, we filtered out the low frequency non-cancer genes and kept high frequency ones for further analysis.

### Function-based screening of gene pairs

The occurrence and progress of cancer are closely related to cell survival, signal transduction, cell growth and death, *etc*. The SL genes were closely associated with cancer, and so, we thought that they played important roles in these cancer-related functions. To further identify SL gene pairs, we applied the genes from the above step for pathway enrichment analysis with DAVID Bioinformatics Resources 6.8^50^. Afterwards, we got the final SL gene pairs and some significant pathways which helped to exploit the identification of SL gene pairs.

### Identification and validation of anticancer drug targets

#### The identification of anticancer drug targets

We assumed that the anticancer drug target was a protein, which could be targeted by at least one anticancer drug. To identify potential anticancer drug targets, we applied the drug-target interactions and drug description information to annotate the identified non-cancer genes in the SL gene pairs we identified above.

#### The validation of the anticancer drug targets

We validated our identified anticancer drug targets with three data sources. Firstly, the human SL gene pair in the SynLethDB database was used. Secondly, the SL gene pair can be validated by Garnett *et al*.’s drug sensitivity experiment results. A SL gene pair could be seen as a specific mutated cancer gene and a drug targeted non-cancer gene. The cell line with the specific mutated cancer gene should have poor survival condition when added drugs to target the SL partner of the specific mutated cancer gene. That is, the cell line was highly sensitive to the drug. Thus, Garnett *et al*.’s drug sensitivity experiment was used be used to validate the anticancer drug target we obtained. Thirdly, text-mining validation was applied to validate our results. For gene G (the non-cancer gene in the SL pair), the number of studies that mentioned gene G in PubMed was K. The number of cancer-related (or SL-related) studies was M. The total number of studies in PubMed was N. By using hypergeometric test, we calculated the probability that at least x of the K articles containing gene G demonstrated that gene G is associated with cancer (or SL).

4 $${P}=1-\sum _{i=0}^{x-1}\frac{(\begin{array}{c}M\\ i\end{array})(\begin{array}{c}N-M\\ K-i\end{array})}{(\begin{array}{c}N\\ K\end{array})}$$

The significance threshold was set to 0.05 and all of the genes with a significant P-value of less than 0.05 were verified to be cancer-related (or SL-related) genes.

## Electronic supplementary material

Dataset 1

Dataset 2

Dataset 3

Dataset 4

## References

[1] Bridges CB (1922). The Origin of Variations in Sexual and Sex-Limited Characters. The American Naturalist.

[2] Hartman JL, Garvik B, Hartwell L (2001). Principles for the buffering of genetic variation. Science.

[3] Dobzhansky T (1946). Genetics of natural populations; recombination and variability in populations of Drosophila pseudoobscura. Genetics.

[4] Lel HH, Friend SH (1997). Integrating Genetic Approaches into the Discovery of Anticancer Drugs. Science.

[5] Chan DA, Giaccia AJ (2011). Harnessing synthetic lethal interactions in anticancer drug discovery. Nature Reviews Drug Discovery.

[6] Canaani D (2014). Application of the concept synthetic lethality toward anticancer therapy: a promise fulfilled?. Cancer Lett..

[7] Srivas R (2016). A Network of Conserved Synthetic Lethal Interactions for Exploration of Precision Cancer Therapy. Mol. Cell.

[8] Farmer H (2005). Targeting the DNA repair defect in BRCA mutant cells as a therapeutic strategy. Nature.

[9] Tutt A (2009). Phase II trial of the oral PARP inhibitor olaparib in BRCA-deficient advanced breast cancer. Journal of Clinical Oncology Official Journal of the American Society of Clinical Oncology.

[10] Robson M (2017). Olaparib for Metastatic Breast Cancer in Patients with a Germline BRCA Mutation. N. Engl. J. Med..

[11] Eskander RN, Tewari KS (2014). PARP inhibition and synthetic lethality in ovarian cancer. Expert Rev. Clin. Pharmacol..

[12] Deshpande R (2013). A comparative genomic approach for identifying synthetic lethal interactions in human cancer. Cancer Res..

[13] Canaani D (2009). Methodological approaches in application of synthetic lethality screening towards anticancer therapy. Br. J. Cancer.

[14] Ferrari E, Lucca C, Foiani M (2010). A lethal combination for cancer cells: synthetic lethality screenings for drug discovery. Eur. J. Cancer.

[15] Wang T (2017). Gene Essentiality Profiling Reveals Gene Networks and Synthetic Lethal Interactions with Oncogenic Ras. Cell.

[16] Ye H, Zhang X, Chen Y, Liu Q, Wei J (2016). Ranking novel cancer driving synthetic lethal gene pairs using TCGA data. Oncotarget.

[17] Conde-Pueyo N, Munteanu A, Sole RV, Rodriguez-Caso C (2009). Human synthetic lethal inference as potential anti-cancer target gene detection. BMC Syst. Biol..

[18] Kranthi T, Rao SB, Manimaran P (2013). Identification of synthetic lethal pairs in biological systems through network information centrality. Molecular bioSystems.

[19] Miller CA, Settle SH, Sulman EP, Aldape KD, Milosavljevic A (2011). Discovering functional modules by identifying recurrent and mutually exclusive mutational patterns in tumors. BMC Med. Genomics.

[20] Srihari S, Singla J, Wong L, Ragan MA (2015). Inferring synthetic lethal interactions from mutual exclusivity of genetic events in cancer. Biol. Direct.

[21] Jerby-Arnon L (2014). Predicting cancer-specific vulnerability via data-driven detection of synthetic lethality. Cell.

[22] Zaman N (2013). Signaling network assessment of mutations and copy number variations predict breast cancer subtype-specific drug targets. Cell reports.

[23] Jones DT, Harris AL (2012). Small-molecule inhibitors of the HIF pathway and synthetic lethal interactions. Expert Opin. Ther. Targets.

[24] Crowder RJ (2009). PIK3CA and PIK3CB inhibition produce synthetic lethality when combined with estrogen deprivation in estrogen receptor-positive breast cancer. Cancer Res..

[25] Morandell S, Yaffe MB (2012). Exploiting synthetic lethal interactions between DNA damage signaling, checkpoint control, and p53 for targeted cancer therapy. Prog. Mol. Biol. Transl. Sci..

[26] Weidle UH, Maisel D, Eick D (2011). Synthetic lethality-based targets for discovery of new cancer therapeutics. Cancer Genomics Proteomics.

[27] Guenther MK, Graab U, Fulda S (2013). Synthetic lethal interaction between PI3K/Akt/mTOR and Ras/MEK/ERK pathway inhibition in rhabdomyosarcoma. Cancer Lett..

[28] Guo J, Liu H, Zheng J (2015). SynLethDB: synthetic lethality database toward discovery of selective and sensitive anticancer drug targets. Nucleic Acids Res..

[29] Bueno R, Mar JC (2017). Changes in gene expression variability reveal a stable synthetic lethal interaction network in BRCA2−ovarian cancers. Methods.

[30] Costa-Cabral S (2016). CDK1 Is a Synthetic Lethal Target for KRAS Mutant Tumours. PLoS One.

[31] Kang J, Sergio CM, Sutherland RL, Musgrove EA (2014). Targeting cyclin-dependent kinase 1 (CDK1) but not CDK4/6 or CDK2 is selectively lethal to MYC-dependent human breast cancer cells. BMC Cancer.

[32] Molenaar JJ (2009). Inactivation of CDK2 is synthetically lethal to MYCN over-expressing cancer cells. Proc. Natl. Acad. Sci. USA.

[33] Nekova TS, Kneitz S, Einsele H, Bargou R, Stuhler G (2016). Silencing of CDK2, but not CDK1, separates mitogenic from anti-apoptotic signaling, sensitizing p53 defective cells for synthetic lethality. Cell cycle (Georgetown, Tex.).

[34] Yang H (2015). Therapeutic target database update 2016: enriched resource for bench to clinical drug target and targeted pathway information. Nucleic Acids Res..

[35] Van dMR, Song HY, Park SH, Abdulkadir SA, Roh M (2014). RNAi screen identifies a synthetic lethal interaction between PIM1 overexpression and PLK1 inhibition. Clinical Cancer Research An Official Journal of the American Association for Cancer Research.

[36] Aarts M (2015). Functional genetic screen identifies increased sensitivity to WEE1 inhibition in cells with defects in Fanconi Anaemia and HR pathways. Mol. Cancer Ther..

[37] Garnett MJ (2012). Systematic identification of genomic markers of drug sensitivity in cancer cells. Nature.

[38] Szczurek E, Misra N, Vingron M (2013). Synthetic sickness or lethality points at candidate combination therapy targets in glioblastoma. Int. J. Cancer.

[39] Wang X, Simon R (2013). Identification of potential synthetic lethal genes to p53 using a computational biology approach. BMC Med. Genomics.

[40] Awan A (2007). Regulatory network motifs and hotspots of cancer genes in a mammalian cellular signalling network. IET Syst. Biol..

[41] Cui Q (2007). A map of human cancer signaling. Mol. Syst. Biol..

[42] Li L (2012). The human phosphotyrosine signaling network: evolution and hotspots of hijacking in cancer. Genome Res..

[43] Gong X (2010). Extracting consistent knowledge from highly inconsistent cancer gene data sources. BMC Bioinformatics.

[44] Futreal PA (2004). A census of human cancer genes. Nat. Rev. Cancer.

[45] Wishart DS (2006). DrugBank: a comprehensive resource for in silico drug discovery and exploration. Nucleic Acids Res..

[46] von Eichborn J (2011). PROMISCUOUS: a database for network-based drug-repositioning. Nucleic Acids Res..

[47] Shannon P (2003). Cytoscape: a software environment for integrated models of biomolecular interaction networks. Genome Res..

[48] Csardi, G. & Nepusz, T. The Igraph Software Package for Complex NetworkResearch. *InterJournal* Complex Systems, 1695 (2006).

[49] Opsahl T (2009). Structure and Evolution of WeightedNetworks. University of London (Queen Mary College), London, UK.

[50] Huang DW (2007). DAVID Bioinformatics Resources: expanded annotation database and novel algorithms to better extract biology from large gene lists. Nucleic Acids Res..

